# Molecular mechanism of anti-cancer activity of phycocyanin in triple-negative breast cancer cells

**DOI:** 10.1186/s12885-015-1784-x

**Published:** 2015-10-23

**Authors:** Mathangi Ravi, Shilpa Tentu, Ganga Baskar, Surabhi Rohan Prasad, Swetha Raghavan, Prajisha Jayaprakash, Jeyaraman Jeyakanthan, Suresh K Rayala, Ganesh Venkatraman

**Affiliations:** 1 Department of Human Genetics, Sri Ramachandra University, Chennai, 600116 India; 2 Department of Biotechnology, Indian Institute of Technology Madras (IITM), Chennai, 600036 India; 3 Department of Bioinformatics, Alagappa University, Karaikudi, India

**Keywords:** Phycocyanin, Anti-neoplastic, Apoptosis, COX-2, MAPK, MDA-MB-231 cells

## Abstract

**Background:**

Triple-negative breast cancers represent an important clinical challenge, as these cancers do not respond to conventional endocrine therapies or other available targeted agents. Phycocyanin (PC), a natural, water soluble and non-toxic molecule is shown to have potent anti-cancer property.

**Methods:**

In this study, we determined the efficacy of PC as an anti-neoplastic agent in vitro on a series of breast cancer cell lines. We studied effects of PC in inducing DNA damage and apoptosis through western blot and qPCR. Also, anti-metastatic and anti-angiogenic properties were studied by classic wound healing and vasculogenic mimicry assays.

**Results:**

We found that triple negative MDA-MB-231 cells were most sensitive to PC (IC50 : 5.98 ± 0.95 μM) as compared to other cells. They also showed decreased cell proliferation and reduced colony formation ability upon treatment with PC. Profile of Cell cycle analysis showed that PC caused G1 arrest which could be attributed to decreased mRNA levels of Cyclin E and CDK-2 and increased p21 levels. Mechanistic studies revealed that PC induced apoptosis as evident by increase in percentage of annexin positive cells, increase in γ-H2AX levels, and by changing the Bcl-2/Bax ratio followed by release of cytochrome C and increased Caspase 9 levels. MDA MB 231 cells treated with PC resulted in decreased cell migration and increased cell adhesive property and also showed anti-angiogenic effects. We also observed that PC suppressed cyclooxygenase-2 (COX-2) expression and prostaglandin E(2) production. All these biological effects of phycocyanin on MDA MB 231 cells could be attributed to decreased MAPK signaling pathway. We also observed that PC is non-toxic to non-malignant cells, platelets and RBC’s.

**Conclusion:**

Taken together, these findings demonstrate, for the first time, that PC may be a promising anti-neoplastic agent for treatment of triple negative breast cancers.

**Electronic supplementary material:**

The online version of this article (doi:10.1186/s12885-015-1784-x) contains supplementary material, which is available to authorized users.

## Background

Cancer of the mammary gland is a complex disease with no known single cause that continues to be a worldwide killer and is the most frequent among cancer related mortality in women [1, 2]. Triple-negative breast cancer, hallmarked by tumors that lack estrogen receptor (ER), progesterone receptor (PR), and HER-2 genes, represent significant clinical challenge as these cancers are highly aggressive and are resistant to conventional endocrine therapy and suffers lack of targeted therapies [3]. Current therapeutic strategies for triple-negative disease include anthracycline/taxane combinations, platinum agents, and other DNA damaging agents. Recently, EGFR targeted therapy has been projected as a single therapeutic mechanism in triple-negative breast cancer, but results were varied [4]. Therefore, there is an increasing need to explore for alternate therapeutic options for these patients.

Therapeutic alternatives originating from food or food supplements appear to be growing in popularity as “nutritional therapy” and are well studied for their chemopreventive and chemotherapeutic effects [5]. Investigations of several food derived bioactive compounds revealed their ability to antagonize dysregulated targets in cellular signaling pathways to exert their anti-neoplastic activities [6]. Thus isolating these bioactive molecules present in marine/land food products, determining their broad range pharmaceutical activity, deducing their specific molecular targets and establishing their minimal toxicity to normal tissues could aid in treatment of cancer [7]. Phycocyanin is an important molecule extracted from the algae *Arthrospira platensis*. It is a natural, water soluble and non-toxic molecule with potent anti-cancer, anti- oxidant and anti-inflammatory properties [8, 9]. Particularly, it is shown to have anti-cancer activity against colon, [10] hepatocellular, [11] cervical [12] and leukemic cell lines [13].

It is widely accepted that critical genes involved in transforming mammary epithelial cells encode proteins that function as dynamic regulators of signal–transduction pathways that regulate cell cycle, differentiation, proliferation and survival [14]. In addition, several groups have established that numerous signaling pathways important for normal breast development are also dysregulated during the progression of breast cancer [15]. Mitogen-activated protein kinase (MAPK) is one such important signaling kinase involved in breast cancer progression [16, 17].

In our study, we studied the role of phycocyanin as an anti-neoplastic agent in triple- negative breast cancer cells for the first time and revealed the molecular mechanism behind its anti-cancer activity.

## Methods

### Materials

PC (Parry India Ltd, India) was dissolved in phenol red free DMEM to prepare a stock solution (200 μM) and stored at 4 °C. Dulbecco’s Modified Eagle Medium (DMEM), fetal bovine serum (FBS) and 0.25 % Trypsin-EDTA were procured from Invitrogen (Carlsbad, CA). Supplements for cell culture were purchased from Sigma Aldrich (St. Louis, MO) and Hi-media. (Mumbai, India) Neutral red was purchased from Sigma Aldrich (St. Louis, MO). Annexin V-FITC Apoptosis Detection Kit was purchased from BD Biosciences (San Jose, CA). The inhibitor cocktail was obtained from Pierce (Rockford, IL) and antibodies against ERK1/2, γH2AX (Ser 139), COX-2, cytochrome C, p-65, AKT and Vinculin were from Cell Signaling Technology (Beverly, MA). Respective secondary mouse, rabbit and goat antibodies were from Santa Cruz Biotechnology (Santa Cruz, CA). Enhanced chemiluminescence solution was from GE health care. Primers for Cyclin E, CDK-2, p-21, Bax, Bcl-2, Caspase 9, Mcl-1,MMP-9,VEGFR-2 and GAPDH were obtained from Eurofins (Luxembourg, Europe). C-DNA reverse transcriptase kit was procured from ABI (Carlsbad, CA) and SYBR green PCR master mix was from Roche (BASEL, Switzerland). Alexa Flour was from Life Technologies (Carlsbad, CA). PGE-2 kit was obtained from Cayman chemicals (Ann Arbor, MI). Matrigel was obtained from BD Biosciences (San Jose, CA, USA).

### Methods

#### Cell lines and cell culture

Human breast cancer cell lines MDA-MB-231 and MCF-7 (procured from NCCS, Pune) SKBR-3, BT-474 and HBL-100 (kindly provided by Dr. Rayala Suresh Kumar, IIT-M) were cultured in high glucose (4.5 g/l) DMEM medium supplemented with 10 % fetal bovine serum,100 units/ml Penicillin, 100 mg/ml Streptomycin, pH 7.4 in 25 cm2, tissue culture flasks at 37 °C under 5 % CO_2_ and 95 % air. Human Breast Normal cell line MCF 10A (a gift from Dr. Annapoorni Rangarajan, IISC, Bangalore) were cultured in DMEM-F12 supplemented with NaHCO_3_ (1.2 g/L), 10 % HS, antibiotic/antimycotic mixture (1 %), EGF (20 ng/ml), HC (500 ng/ml), Cholera Toxin (100 ng/ml) and were maintained at 37 °C in 5 % CO_2_,95 % air and passaged thrice a week.

### PC treatment

Initially a dose–response study was carried out to determine the suitable dose for inhibition of cell growth and induction of apoptosis. Accordingly, the dosage and time points varied between parameters studied and are described in the respective methods.

### Neutral red staining assay

Cells 2 x 10^4^ were seeded in 96-well plates a day prior to treatment. Cell viability for the treated cells (1-20 μM/PC) was examined after 24 h by using neutral red assay as outlined by Guillermo et al. [18]. Briefly, post drug treatment neutral red (40 μg/ml) was added and after 2 h incubation at 37 °C the plate was washed and the dye was extracted with acidified ethanol solution. Absorbance was read at 540 nm and cell viability was ascertained by measuring the absorbance of the treated cell and comparing it to untreated negative controls.

### Clonogenic assay

500 viable cells were seeded in 60 mm plates and were allowed to attach overnight. Cells were incubated with 1, 3 and 5 μM/PC for 24 h and then incubated for an additional 10 days in complete medium to allow colonies to form. The colonies obtained were washed with PBS, fixed in methanol, followed by staining with crystal violet. Experiments were done in triplicate. The colonies were counted and compared to controls.

### Scratch assay /In vitro cell migration assay

5 x 10^5^ cells/well were grown on 6 well plates to form confluent monolayer. Gaps were created in confluent cell layers using micropipette tips, followed by a rinse with PBS. Cells were treated with 3 μM/PC and as control, cells growing in complete media were maintained. The wound closure was monitored by phase-contrast microscopy and photographed at the 0th and 24th hour. The percentage of migration was calculated using the formulae

$$ \mathrm{PercentageMigration}=\frac{\kern.09em \mathrm{Widthofthewound}\kern0.28em \mathrm{at}\kern0.28em 0\mathrm{h}-\mathrm{Width}\kern0.28em \mathrm{of}\kern0.28em \mathrm{the}\kern0.28em \mathrm{wound}\kern0.28em \mathrm{at}\kern0.28em 24\mathrm{h}}{\mathrm{Width}\kern0.28em \mathrm{of}\kern0.28em \mathrm{the}\kern0.28em \mathrm{wound}\mathrm{at}0\mathrm{h}}*100 $$

### Hanging drop aggregation assay

Cells grown to sub confluence in culture plates were exposed to 3 μM/PC for 24 h and harvested. The cells were re-suspended in complete media and 20 μL of the cell suspension containing approximately 5000 cells was placed onto the inner surface of the lid of the petri dish, which was then placed on the petri dish containing serum free media (such that the drops hang from the lid with the cells suspended within them). After overnight incubation at 37 °C the lid was inverted, cells were pipetted 10 times and images were documented using 10X objective of a phase contrast microscope.

### Actin dynamics study using confocal microscopy

Cells were plated onto glass coverslips in culture dishes and allowed to attach overnight. After a 24 h treatment to 3 μM/PC cells were fixed for 20 min in 1 % paraformaldehyde (PFM) followed by incubation in 0.1 M glycine solution for 5 min. Cells were permeabilized using 0.1 % Triton X 100 for 3–5 min. A series of PBS rinse was given after which 5U of phalloidin was added and incubated in dark for 45 min. Cells were counter stained with propidium iodide (300 μg/mL) and mounted on DPX and observed under the confocal microscope at 488 nm.

### Cell cycle analysis by flow cytometry

Cells (2 x 10^5^) were seeded in 60 mm plates and synchronized by culturing them in serum free medium. After 24 h treatment with 5 μM/PC both adherent and floating cells were collected, washed twice with cold PBS, and stained with hypotonic propidium iodide solution (50 μg/ml PI, 40 μg/ml RNAse A, 0.3 μl/ml Triton X 100 in 0.1 % trisodium citrate) for 15 min. Stained cells were analyzed by flow-cytometry using BD Accuri C6 (Becton Dickinson, San Jose, CA) at 488 nm capturing 20,000 events.

### Annexin V PE and 7AAD staining

Cells (1 × 10^6^) were seeded in 100 mm plates and allowed to attach overnight. Next cells were treated with 1 μM (IC25) and 5 μM (IC50) PC for 6 h. Following treatment cells were harvested and stained using Annexin V-phycoerythrin (PE) apoptosis detection kit (BD Pharmingen) according to the manufacturer’s recommendations. Briefly, the cells were trypsinized, washed in PBS, and resuspended in binding buffer (1 × 10^6^/mL). Equal volumes of Annexin V-PE and 7-amino-actinomycin D (7-AAD) were added and incubated for 15 min at room temperature in the dark and analyzed by flow-cytometry.

### RNA isolation and real-time reverse transcription-PCR

Cells (1 x 10^6^) were seeded in 100 mm plates and allowed to attach overnight. Next cells were treated with 5 μM/PC for cell cycle and apoptotic related gene expression studies and 3 μM/PC for migration and angiogenesis related gene expression studies for 24 h. Total RNA was isolated from untreated and drug-treated cells using Trizol reagent. The yield and purity of isolated RNA was checked by UV spectrophotometry. 2 μg of total RNA were used in reverse transcription reactions using the C-DNA reverse transcriptase kit according to manufacturer’s protocol. Gene expression in untreated and PC - treated cells were determined by real-time PCR using the reverse transcription product, gene-specific primers (5 pmol) and SYBR green in a 20 μL reaction volume on the 7500 Fast RT PCR machine (ABI, Carlsbad, CA). Relative changes in mRNA expression levels were assessed by the 2^-ΔΔCT^ method and changes in mRNA expression of the target gene were normalized to that of GAPDH gene. The primer pairs of the selected genes are listed in Table 1.

**Table 1:** List for primer pairs used in qPCR

Gene	Orientation	Primer sequence 5′ TO 3′	Accession number
CYCLIN E	F	TCAGGGTATCAGTGGTGCGA	XM_005259370.2
	R	CAAATCCAAGCTGTCTCTGTG	
CDK-2	F	TTTGCTGAGATGGTGACTCG	NM_001798.4
	R	CTTCATCCAGGGGAGGTACA	
P21	F	AAG ACC ATG TGG ACC TGT	NM_001291549.1
	R	GGT AGA AAT CTG TCA TGC TG	
BAX	F	TGCTTCAGGGTTTCATCCAG	NM_001291428.1
	R	GGCGGCAATCATCCTCTG	
Bcl-2	F	AGGAAGTGAACATTTCGGTGAC	NM_000633.2
	R	GCTCAGTTCCAGGACCAGGC	
CASPASE 9	F	CCAGAGATTCGCAAACCAGAGG	XM_011542273.1
	R	GAGCACCGACATCACCAAATCC	
MCL1	F	GGGCAGGATTGTGACTCTCATT	NM_001197320.1
	R	GATGCAGCTTTCTTGGTTTATGG	
COX-2	F	GATACTCAGGCAGAGATGATCTACCC	NM_000963.3
	R	AGACCAGGCACCAGACCAAAGA	
VEGFR2	F	TGCCTACCTCACCTGTTTC	NM_002253.2
	R	GGCTCTTTCGCTTACTGTTC	
MMP-9	F	ACCTCGAACTTTGACAGCGAC	NM_004994.2
	R	GAGGAATGATCTAAGCCCAGC	
GAPDH	F	ACCCAGAAGACTGTGGATGG	NM_001289746.1
	R	CAGTGAGCTTCCCGTTCAG	

### Protein extraction, SDS-PAGE & Western blot analysis

Cells (1 × 10^6^) were seeded in 100 mm plates and allowed to attach overnight and treated with 3 μM/PC at time points described in figures. For whole cell protein, lysates were collected using RIPA buffer containing protease/phosphatase inhibitor cocktail. For nuclear protein, first cytoplasmic extract was collected using buffer A (1 M HEPES pH 7.9, 2 M KCL,o.5 M EDTA pH 8, 0.1 M EGTA pH 7, 0.1 M DTT, 10 % NP40, protease inhibitor) and later nuclear protein extract were collected using buffer B(1 M HEPES pH 7.9, 5 M Nacl,o.5 M EDTA pH 8, 0.1 M EGTA pH 7, 0.1 M DTT and protease inhibitor). The protein concentrations were measured using Bio-Rad protein estimation kit, spectrophotometrically. Equivalent amounts of protein lysates were separated by electrophoresis in 12 % SDS polyacrylamide gel and blotted onto nitrocellulose membrane. The blotted membranes were incubated with different primary antibodies, followed by incubations with secondary antibodies. The proteins were visualized using an ECL kit.

### PGE2 assay

Amounts of PGE2 in the conditioned media collected from the arachidonic acid stimulated (10 μM) PC-treated (5 μM/PC) cells were determined by an enzyme-linked immunosorbent assay according to the instructions of the manufacturer (Cayman chemicals).

### Assay for vasculogenic mimicry

A 96-well tissue culture plate was evenly coated with 50 μl/well growth factor-reduced matrigel, which was allowed to solidify at 37 °C for 45 min. The cell suspension containing 3 μM/PC was added (200 μl /well) onto the surface of the matrigel and incubated at 37 °C. Cells were monitored through 2-10 h and photographed using a phase contrast microscope.

### Platelet aggregation assay

Briefly, 1 mL of whole blood was collected in a heparin vaccutainer and spun. Platelets were concentrated in 1 mL of RPMI 1640 and treated to 20 μM/PC, the highest dose used in initial testing. PBS and collagen were maintained as negative and positive controls respectively. Following 10 min incubation a drop of the cell suspension was placed on glass slides and observed for any aggregation under 10× magnification of a phase contrast microscope.

### Hemolytic assay

Briefly, 1 mL of whole blood was collected in a heparin vaccutainer and spun. The RBC pellet was re-suspended in 1 mL of RPMI 1640 to which 20 μM/PC was added. RBC’s exposed to 0.1 % Triton X 100 were maintained as a positive control. After 2 h incubation at 37 °C, cells were spun and re-suspended in PBS. A drop of the cell suspension was placed on glass slides and observed for any morphological changes under 40× magnification of a phase contrast microscope.

### Statistical analysis

All the experiments were performed at least three times, independently. The data obtained were expressed as ‘mean ± standard deviation’. Wherever appropriate, the data were subjected to Student’s *t*-test using Graph Pad Prism Version 5.0. A value of *p* < 0.05 was considered as significant.

## Results

### PC inhibits proliferation and colony formation ability of breast cancer cells

**Fig. 1:**
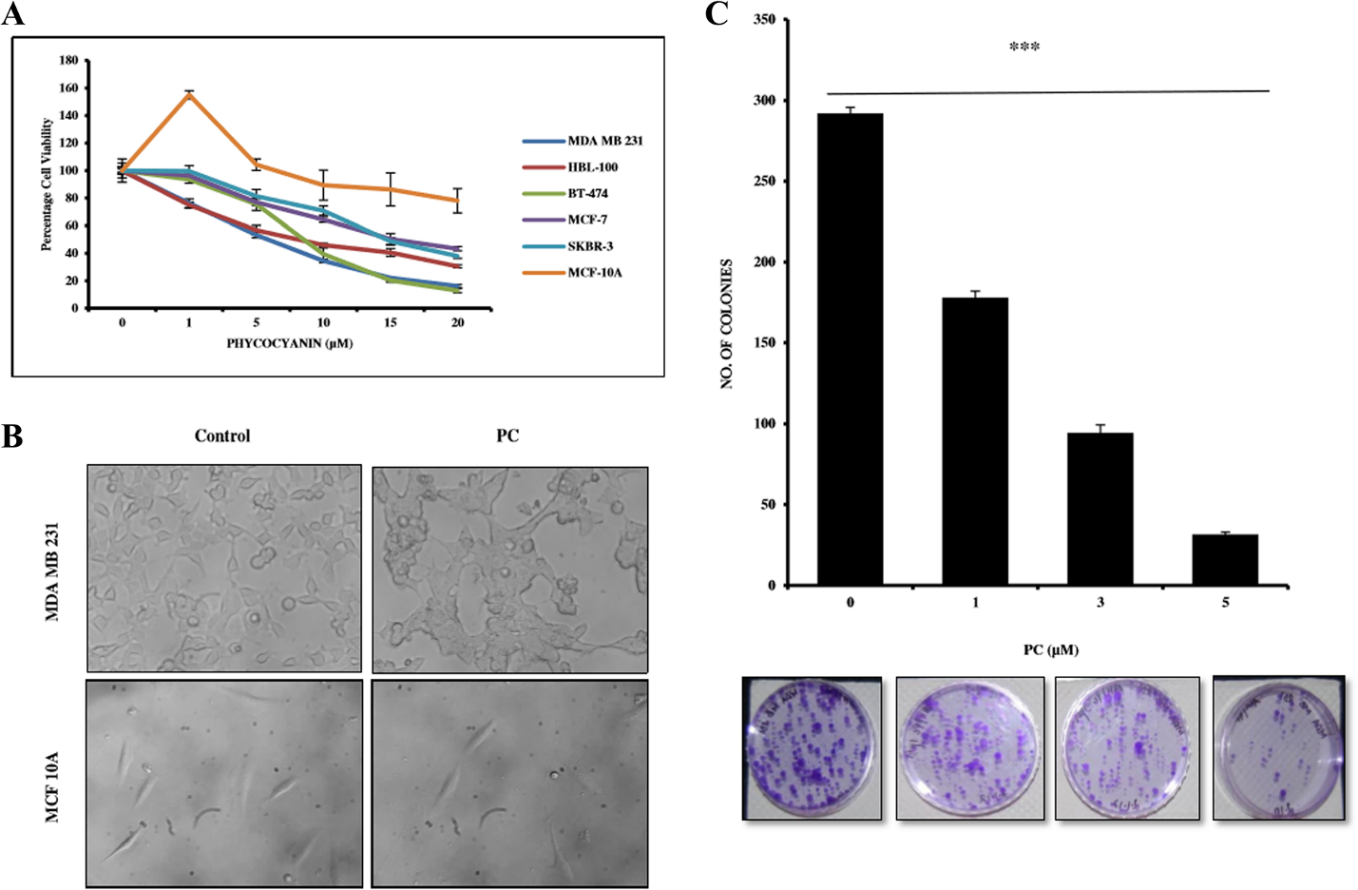
Growth inhibitory effects of Phycocyanin on breast cancer cells. **a** The anti-proliferative effects of phycocyanin on a panel of breast cancer cells and MCF10A cells were analyzed by neutral red staining assay. Data are expressed as mean ± SD; (*n* = 3). **b** Representative micrographs from random fields of view (magnification 20X) of indicated cells treated with and without phycocyanin. **c** Phycocyanin at different concentrations inhibits clonogenecity of MDA MB 231 cells as determined by clonogenic cell survival assay. Quantitative representation of reduction in number of colonies with representative images. ***P < 0.0001 compared with untreated controls

Screening for anti-cancer effects of PC by neutral red uptake assay on breast cancer cells representing different molecular subtypes and on Normal Human Breast cells showed that PC inhibited proliferation of breast cancer cells in a dose dependent manner. The IC50 values for the tested panel of breast cancer cells ranged between 5 and 15 μM (Table 2). It was interesting to note that triple negative MDA MB 231 cells were found to be most sensitive to PC with an IC50 of 5.98 μM in as early as 24 h and hence used as a representative cell line for further experiments (Fig. 1a). There is no significant effect of PC on normal Human Breast MCF 10A cells indicating its non-toxicity to normal cells. Upon treatment with PC, we observed a change in cell morphology -comparable to morphological changes associated with apoptosis (Fig. 1b).

**Table 2:** IC50 values

Cell line	IC_50_ (μM) 24H
Breast cancer cell lines	
MDA MB 231	5.98 ± 0.95
HBL 100	8.31 ± 1.59
BT-474	8.45 ± 0.94
MCF-7	15.43 ± 2.11
SKBR-3	15.73 ± 0.74
Normal cells	
MCF10A	>20

Further to establish the inhibitory role of PC on transforming properties of cancer cells, we performed clonogenic assay. Results showed that PC treated cells showed significant reduction in colony formation when compared to controls, indicative of potent inhibition of cell growth and reproductive integrity (Fig. 1c).

### PC inhibits wound healing and migration of MDA MB 231 breast cancer cells

**Fig. 2:**
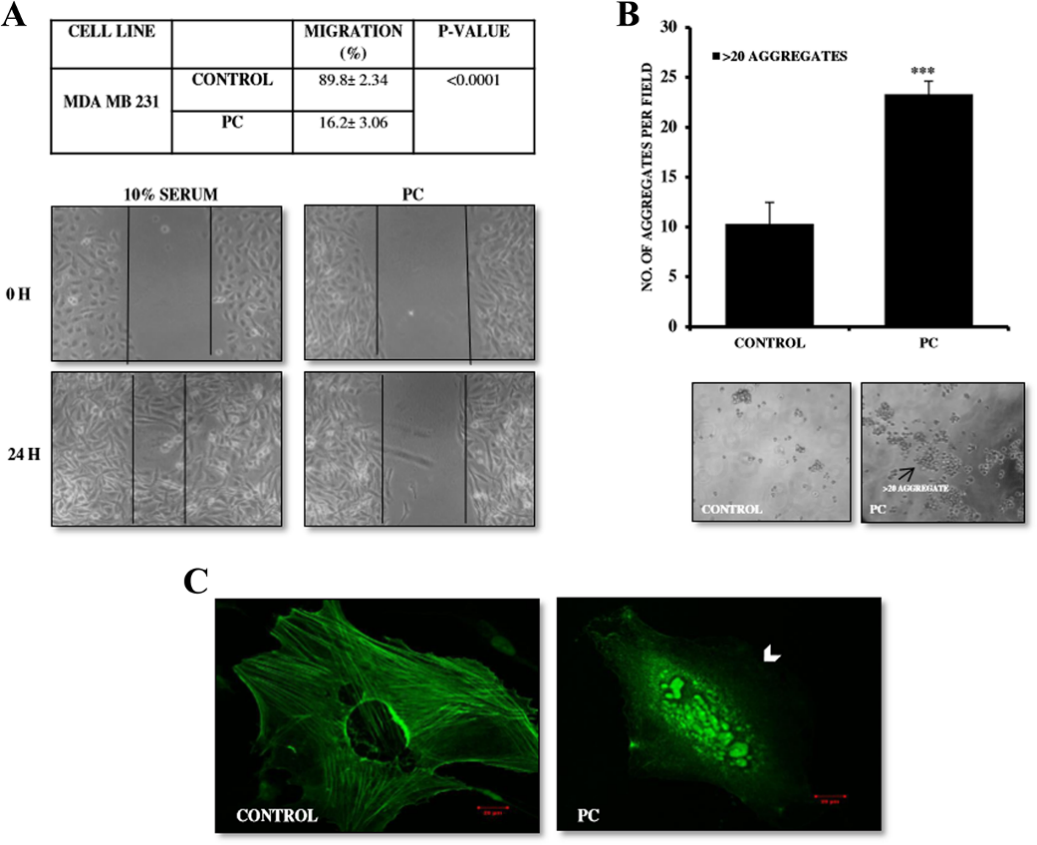
Phycocyanin inhibits cell migration in MDA MB 231 cells. **a** Percentage of cell migration into the wound scratch with and without treatment with PC was quantified and compared against that of controls. Representative images of wound healing at 0 and 24 h following scratch induction and PC treatment. **b** Assessment of cellular aggregation by hanging drop aggregation assay showed increased cell-cell adhesion (>20 aggregates) in PC treated MDA MB 231 cells (arrows indicate >20 aggregates). (***P < 0.0001 compared with untreated controls) (**c**) Confocal scanning microscopy analysis for phalloidin in MDA MB 231 cells showed microfilament network collapse after PC treatment

Reduced clonogenecity is usually associated with loss of invasion capabilities of tumor cells [19]. Since PC treated cells showed a significant reduction in colony formation ability, we next sought to determine the effects of PC on the migration behavior of breast cancer cells. Classic wound healing assay results showed that PC treated cells showed decreased wound healing in comparison to control. The percentage of wound closure in PC treated group decreased to 16.2 ± 3.06 % Vs 89.8 ± 2.34 % in the control group (Fig. 2a). Further, we determined the effect of PC on the phenotypic characteristics associated with metastatic activity by hanging drop aggregation assay. Results showed that there is an increased adhesiveness with > 20 aggregates/field in PC treated group. The average aggregates per field with a 3 μM dose of PC were 23.3 ± 1.3 Vs 10.3 ± 2.15 in control (Fig. 2b). Additionally, this disruption of cellular motility was microscopically analyzed by phalloidin stain to visualize actin filaments. As indicated by arrow head, PC treated cells showed collapsed actin cytoskeleton when compared to the untreated control (Fig. 2c). Collectively these results suggest that PC could inhibit cell migration via cytoskeleton disruption and also confer adhesiveness to cells, thereby playing an important role in suppressing invasion.

### PC induces G0/G1 cell cycle arrest of MDA MB 231 breast cancer cells

Since PC inhibited cell proliferation, we further determined to assess the role of PC in cell cycle progression of MDA MB 231 cells by flow cytometry. Results show that PC induced significant G0/G1 cell cycle arrest. In comparison to untreated controls, there is an increase in percentage of cells in G0/G1 phase (62.1 ± 1.1 % Vs 73.2 ± 0.2 %) with a concomitant decrease in the percentage of cells in S (18.4 ± 1.1 % Vs 14.3 ± 0.04 %) and G2-M phases (17.7 ± 3.5 % Vs 10.7 ± 0.4 %) of the cell cycle (Table 3).

**Table 3:** DNA content analysis

CELL LINE		SUBG0/G1 (%)	G0/G1 (%)	S (%)	G2-M (%)
MDA MB 231	CONTROL	2.4 ± 1.6	62.1 ± 1.1	18.4 ± 1.1	17.7 ± 3.5
	PC-IC50	1.7 ± 0.3	73.2 ± 0.2	14.3 ± 0.04	10.7 ± 0.4

**Fig. 3:**
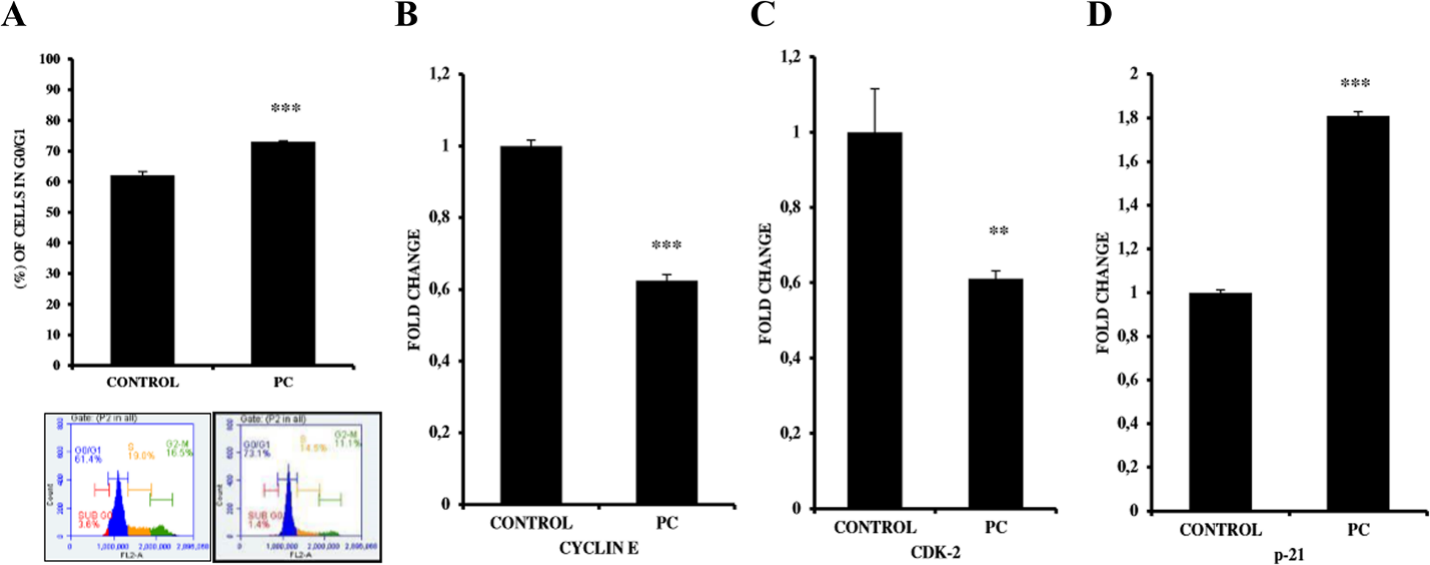
Phycocyanin induces G1 phase arrest in MDA MB 231 cells. **a** CeIl cycle analysis showing increased accumulation of cells in G0/G1 phase when treated to phycocyanin with representative histograms. qPCR analysis for genes involved in G1 phase arrest (**b**) Cyclin E (**c**) Cdk2 and (**d**) p-21. GAPDH was used as internal control for normalization. (***P < 0.0001, ** P < 0.002 compared with untreated controls)

With PC treatment, there is an increase of about 1.17 fold in number of cells in G0/G1 phase as compared to untreated controls, suggesting the role of PC in inhibiting entry into S-phase (Fig. 3a). Further to establish the fact that PC inhibits S-phase entry, we tested for the levels of cell cycle regulatory proteins involved in G1 to S transition -Cyclin E, CDK2, and CDK inhibitor p21 by qPCR analysis. We observed that mRNA levels of both Cyclin E and CDK2 decreased by 1.60 and 1.64 fold respectively and of p21 increased by 1.81 fold in comparison to untreated control, strongly indicating the triggering of G1 arrest and blocking S phase entry (Fig. 3b-d).

### PC induces apoptosis of MDA MB 231 breast cancer cells

**Fig. 4:**
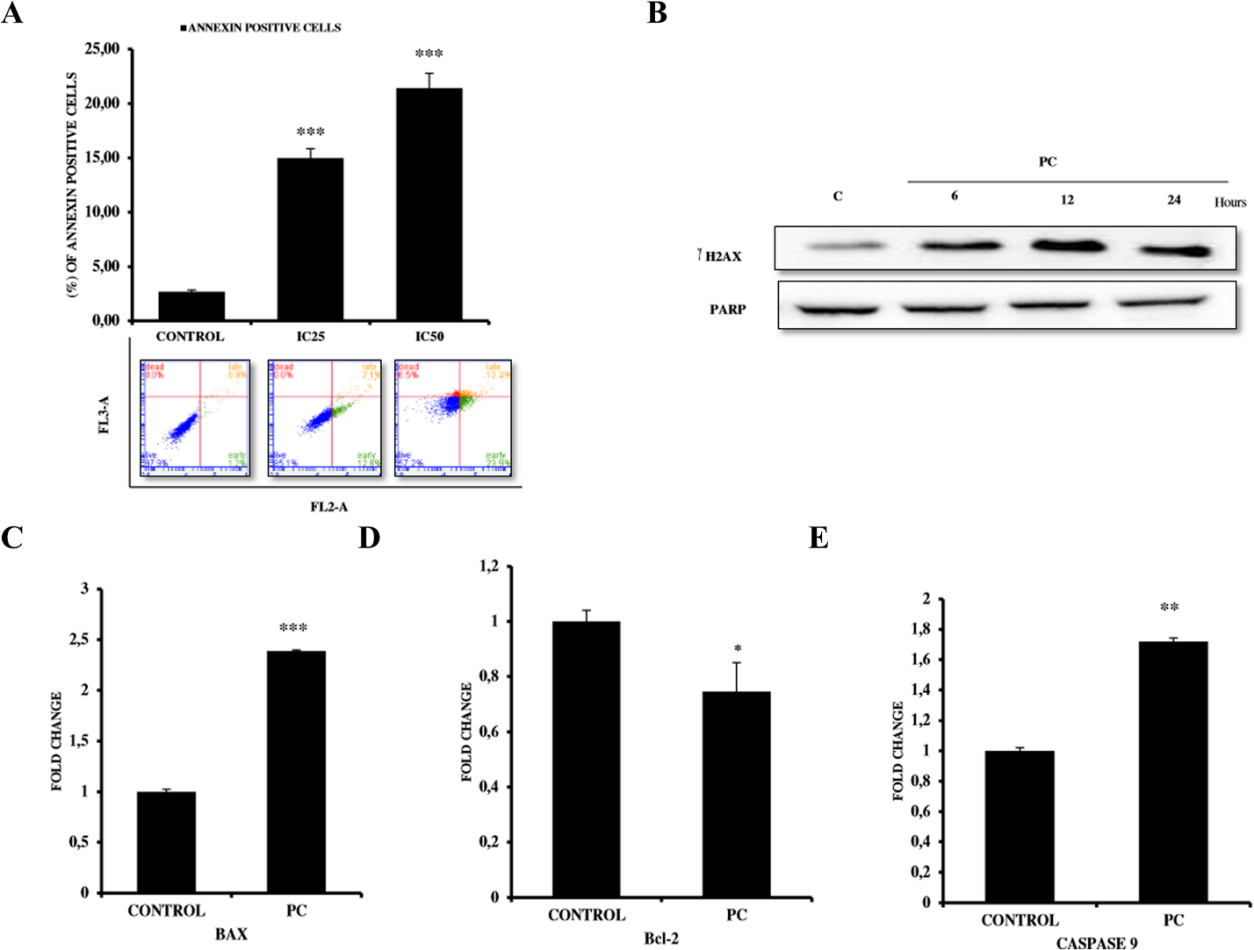
Phycocyanin induces apoptosis in MDA MB 231 cells. **a** Quantitative representation of early apoptotic cells at IC25 &50 doses at the end of 6 h treatment with PC as analyzed by PE annexin V and 7 AAD staining with representative scatter plots showing shift of cells from live to early apoptosis. **b** Western blot analysis for phospho-H2AX (C) qPCR analysis of apoptotic markers in MDA MB 231 cells. qPCR analysis for (**c**) BAX, (**d**) Bcl-2 and (**e**) caspase 9. GAPDH was used as internal control for normalization (***P < 0.0001, ** P = 0.001, *P = 0.01 compared with untreated controls)

As PC is known to induce apoptosis in cancer cells [8, 9, 13, 20], we next determined to study the extent of apoptosis in MDA MB 231 cells by Annexin V PE and 7AAD staining. Results showed that PC treated MDA MB 231 cells demonstrated a high induction of apoptosis in comparison to untreated controls. The percentage of apoptotic cells increased gradually from 2.69 % in untreated controls to 14.99 % and 21.43 % in IC25 and IC50 treated cells with a fold increase of 5.57 and 7.96 respectively (Fig. 4a and Table 4). Consistent with this, results from western blot analysis for phospho-H2AX (γH2AX) revealed a dose dependent increase in γH2AX levels upon treatment with PC - an indicative of accumulating DNA double stranded breaks (Fig. 4b). Further to gain a deeper insight into the mechanism of apoptosis induced by PC, we next tested the release of cytochrome C - an important initiator step for the activation of caspases. Western blot results for cytochrome C revealed a sustained release of cytochrome C from 1.5 h onwards with maximal release at 6 h, indicating initiation of apoptosis (Additional file 1: Figure S1). Consistently, qPCR analysis for apoptotic markers like Bax, Bcl2 and caspases 9 in PC treated cells showed that there is a change in the Bcl-2/Bax ratio (Increased Bax (2.38 fold higher) to Bcl-2 (1.34 fold lower) ratio) and a 1.72-fold increase in Caspase 9 levels in comparison to untreated controls (Fig. 4 c-e) which was supported by Western blot analysis for cleaved caspase 9 (Additional file 2: Figure S2). Taken together these results suggest that PC is cytotoxic and induces apoptosis in MDA MB 231 cells in a dose and time dependent manner.

**Table 4:** Percentage of cells undergoing apoptosis

MDA MB 231	Live	Early apoptotic	Late apoptotic	Dead
CONTROL	96.39 ± 0.72	2.69 ± 0.26	0.91 ± 0.43	0.01 ± 0.02
PC - IC25	83.65 ± 1.47	14.99 ± 1.49	1.34 ± 0.25	0.01 ± 0.02
PC - IC50	59.18 ± 1.89	21.42 ± 2.30	12.24 ± 0.77	7.15 ± 0.85

### PC targets MAPK signaling in MDA MB 231 breast cancer cells

**Fig. 5:**
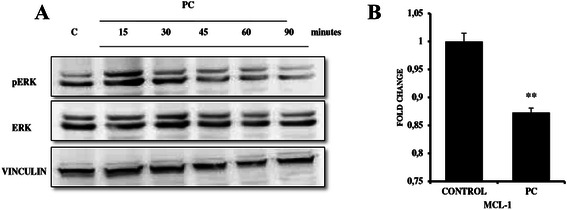
Phycocyanin targets MAPK signaling pathway. **a** MDA MB 231 cells were treated with 3 μM concentration of PC at the indicated time points and phospho ERK (P42/44 MAPK) and total ERK levels were analysed by Western blotting. **b** qPCR analysis for MCL-1 gene expression in MDA MB 231 cells treated with or without PC. (** P = 0.009 compared with untreated controls)

We next aimed to determine the specific signaling pathway targeted by PC to induce apoptosis in MDA MB 231 breast cancer cells. We screened for several signaling pathways that are likely to be involved in cell growth, proliferation, and survival of TNBC’s. [21] Results show that PC treatment did not alter the phosphorylation levels of both AKT and NFkB-p65, (Additional file 3: Figure S3A and S3B) whereas a significant decrease in the phosphorylation of ERK1/2 was observed in as early as 30 min following treatment with maximal decrease at 90 min (Fig. 5a). Consistently, we also observed a significant (1.14 fold) decrease in the mRNA levels of MCL-1 - a downstream target of ERK1/2 signaling and a key regulator for TNBC survival (Fig. 5b).

### PC inhibits COX-2 expression in breast cancer cells

**Fig. 6:**
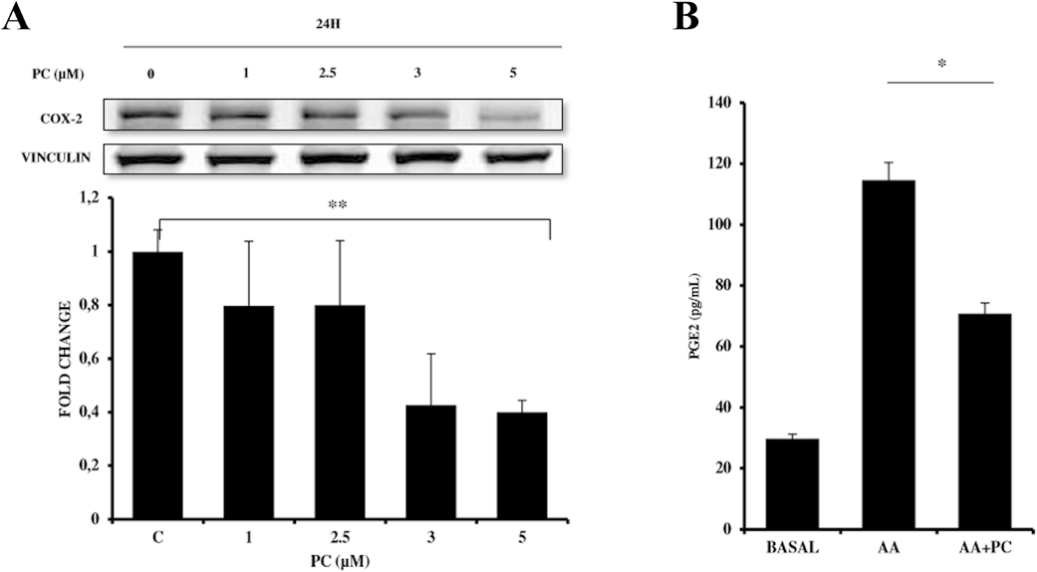
Phycocyanin suppressed COX-2 expression and PGE2 production. MDA-MB-231 cells were treated with phycocyanin at indicated concentrations for 24 h and (**a**) COX-2 protein and mRNA levels were analyzed by Western blotting and qPCR respectively. **b** PGE2 levels in arachidonic acid stimulated and phycocyanin treated MDA-MB-231 cells as analyzed by ELISA. (** P = 0.004 compared with untreated controls)

Since TNBC’s are known for high expression of COX-2 and COX-2 downregulation plays a key role in apoptosis [22]. We next determined the levels of COX-2 protein and mRNA by Western blot and qPCR, respectively. Results showed that PC treated cell showed a decrease in COX-2 protein and mRNA levels (Fig. 6a). Consistently, we also observed a decrease in PGE2 production (a downstream product of COX-2) in arachidonic acid stimulated MDA-MB-231 cells upon treatment with PC. The basal level of PGE2 released in MDA MB 231 cells was 29.7 pg/ml. Upon stimulation with 10 μM arachidonic acid for 18 h and followed by treatment to PC for 24 h, there was a decrease in the level of PGE2 from 114.6 pg/ml to 70.7 pg/ml (Fig. 6b).

### PC inhibits channel formation of breast cancer cells

**Fig. 7:**
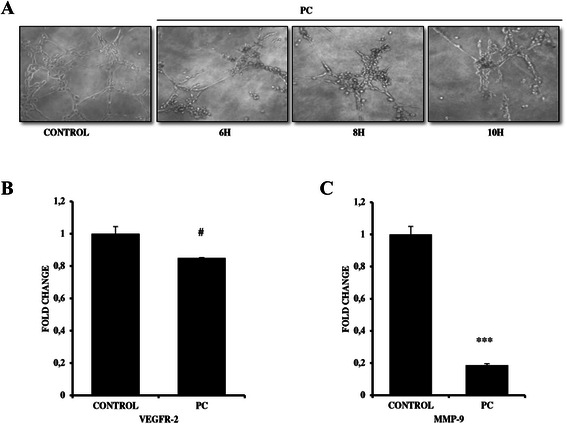
Phycocyanin inhibits vascular channel formation in MDA MB 231 cells (**a**) Representative phase contrast images of vascular channels monitored through 0–10 h following treatment with 3 μm PC. Inhibition of vascular channel formation was evident in as early as 6 h indicating potent anti-angiogenic effects (**b**) qPCR analysis for VEGFR2 and MMP-9 genes in MDA MB 231 cells treated with 3 μM or without PC for 24 h. (^*#*^ P = 0.064, ***P < 0.0001 compared with untreated controls)

It is known that high COX-2 expression in TNBCs promotes increased angiogenesis which is in turn mediated by key regulators like VEGFR2 and MMP-9 [23]. Based on this, we next sought to determine the kinetics of vascular channel formation in MDA MB 231 cells upon treatment with PC by vasculogenic mimicry assay. Results showed that 3 μM dose of PC significantly inhibited the number of vascular channels formed as compared with untreated control cells (Fig. 7a). Subsequently, qPCR analysis showed a significant decrease in the mRNA levels of VEGFR2 and MMP-9 by 1.17 and 5.55 fold, respectively (Fig. 7b-c). Therefore failure of cells to form patterned networks on matrigel and decrease in transcription of genes involved in regulating angiogenesis endorses the antiangiogenic potential of PC.

### PC is non-toxic to blood cells

We next determined the PC’s compatibility to RBC’s and platelet aggregation inhibitory effect by in vitro hemolytic assay and aggregation assay. Results showed no aberrant morphological changes to RBC’s and showed inability to cause platelet aggregation upon treatment with PC- indicating that PC is safe to use for therapy (Additional file 4: Figure S4A and S4B).

## Discussion

It is well known that breast cancer is not the one form of cancer. Gene expression profiling has revealed five molecular subtypes and diagnosis is based upon the presence or absence of hormone receptor-related genes ER, PR and HER2 [24]. Triple-negative breast cancer is one such subtype that is clinically negative for these three receptors and accounts for 10–17 % of all breast carcinomas [25]. It is characterized by its highly aggressive behavior, increased metastasis, poor prognosis and lack of targeted therapies - thus intensifying the need for identifying novel therapeutic strategies for this subset of patients.

Over the last few decades application of natural products for chemoprevention/therapy has gained importance [26]. More recently, pharmacologically active marine derived compounds have been shown to have potent anti-cancer activity with little or no toxic side effects [7, 27]. Induction of apoptosis is the key mechanism by which cytotoxicity is achieved with these compounds [28]. One such compound is the biliprotein PC from the edible *Arthrospira platensis* which in the recent past has been investigated for its anticancer effects on solid malignancies [29]. To the best of our knowledge, this is the first study to demonstrate the anti-cancer effect of PC on breast cancer cell - in particular TNBC cell line. The study also highlights the mechanism underlying PCs cytotoxic, anti-metastatic and anti-angiogenic effects. Our study clearly demonstrated that PC selectively targets MAPK signaling pathway and it also altered the expression of proteins involved in cell cycle and cell survival by which it mediates its growth inhibitory and apoptosis.

Since tissue invasion and metastasis are the main causes for mortality in triple negative breast cancer [30], we investigated the effects of PC on cellular migration and aggregation behavior of MDA MB 231. Our results demonstrated that PC treatment has a direct effect on the aggressive behavior of cells and is evident by decreased migration potential associated with disruption of actin microfilaments and increased aggregative property.

It is well known that cyclin dependent kinases play crucial role in regulation of cell cycle progression. Deregulated activity of these kinases contributes to increased cellular proliferation which has been reported in a wide variety of human cancers [31]. Our initial screening results indicated that PC inhibits proliferation of MDA MB 231 cells and its inhibition correlated well with a decrease in the expression levels of Cyclin E and CDK-2, which are required to mediate the G1-S transition. We also observed an increase in the levels of tumor suppressor p21 that disables the cells to start DNA synthesis, thus confirming arrest at G1/S boundary.

Decrease in CDK-2 levels induces sustained DNA damage and G1 arrest thereby pushing cells to enter apoptosis [31]. It is accepted that transformed cells acquire the ability to breach this dogma and fail to enter the apoptotic pathway resulting in uncontrolled proliferation. In our study, we observed significant induction of apoptosis in PC treated breast cancer cells indicative of PC‘s ability to activate the apoptotic pathway.

Arrest of cell cycle and activation of apoptosis are cellular responses to DNA damage [32]. Since TNBCs are characterized by genomic instability as a consequence of double-stranded DNA repair deficiency, DNA alkylating agents are currently used as conventional therapeutic agents [33–35]. Moreover, DNA damaging agents are more effective against rapidly proliferating cells thus making cancer cells more susceptible than their normal counterparts [36]. Therefore, the focus is on dietary phytochemicals which could trigger damage to cellular DNA, thereby accelerating cell death. We observed a pronounced increase in γ-H2AX in a time-dependent manner following PC treatment which clearly correlated with cell cycle arrest and increased apoptosis.

It is widely accepted that apoptosis is controlled by the Bcl-2 family members. Altered expression of the Bcl-2 family of proteins: Bax and Bcl-2 resulting in changing the Bcl-2/Bax ratio is frequently implicated in breast cancers and is often associated with poor survival [37–41]. As reported previously in other cancers types [12, 42], we also observed a change in the Bcl-2/Bax ratio with increase in cytochrome C and caspase 9 levels with PC treatment.

Most tumor cells show increased MAPK signaling by which they survive and avoid undergoing apoptosis [43]. In our study we observed PC downregulated ERK1/2 signaling, which could be one of the reason for pushing cells from survival to apoptosis. This decreased phosphorylation of ERK1/2 correlated with decreased expression of Mcl-1, an emerging target for treatment of chemoresistant TNBC. Taken together these results suggest that PC induced apoptosis in MDA MB 231 cells through inactivation of ERK pathway, and inhibition of MCL-1.

The biological function of COX-2 is to convert arachidonic acid into prostaglandins. This inducible enzyme is deregulated in many human tumors including tumors of the breast and plays a key role in tumor progression and chemoresistance [44, 45]. Several pre-clinical studies using celecoxib have shown anti-tumour capacities linked with induction of apoptosis and activation of caspases following COX-2 inhibition [46, 47]. Reduction in COX-2 expression and PGE-2 production levels confirmed apoptosis induction in PC treated cells.

Highly invasive and metastatic breast cancer cells with high COX-2 levels form patterned vascular channels on matrigel. PGE-2 a tightly regulated product of COX, behaves as a mitogen facilitating angiogenisis [48]. Our study also showed that PC via inhibiting COX-2 levels decreased anti-angiogenic effects in TNBCs. Another important highlight of this study is that PC did not cause significant toxicity to normal cells and blood cells, suggesting that PC possess selectivity between cancerous and noncancerous cells.

## Conclusion

**Fig. 8:**
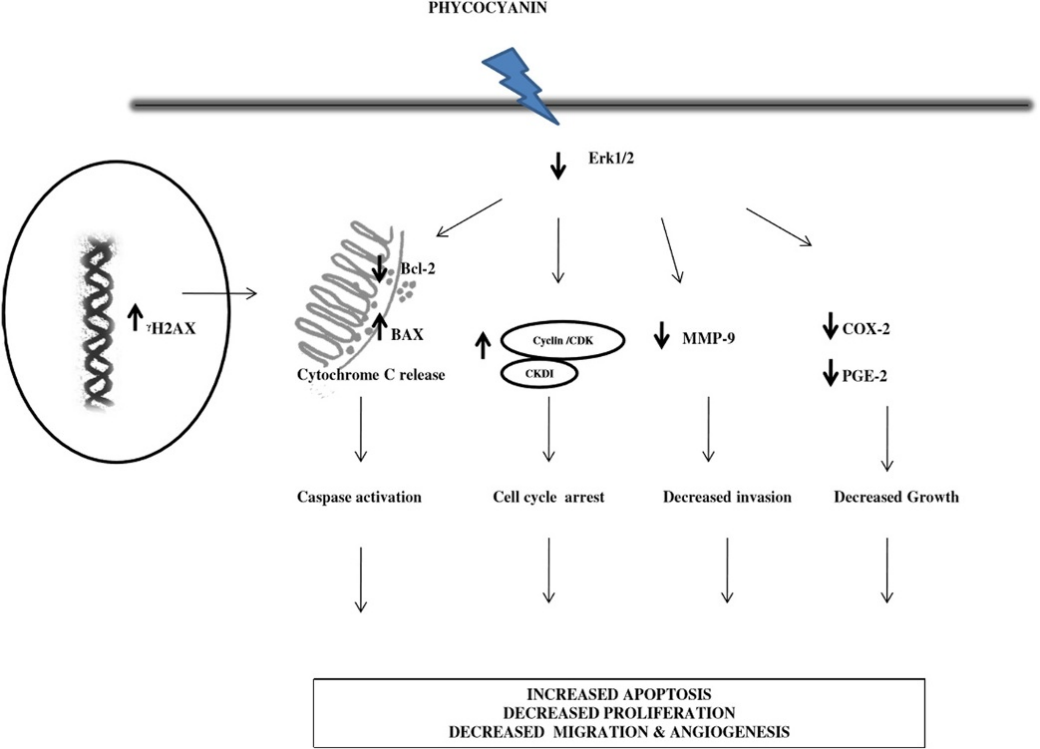
Schematic representation of PC action in MDA MB 231 cells. PC in MDA MB 231 cells (i) inhibits the proliferation (ii) inhibits metastasis via actin cytoskeleton disruption (iii) promotes change in the Bcl-2/Bax ratio (iv) suppresses angiogenesis and (v) down-regulates MAPK signaling pathways to elicit cell death

In conclusion, this study demonstrated that PC in TNBC cells (i) inhibits the proliferation (ii) promotes change in the Bcl-2/Bax ratio (iii) inhibits metastasis via actin cytoskeleton disruption (iv) suppresses angiogenesis and (v) down-regulates MAPK signaling pathways to elicit cell death (Fig. 8). This study proposes that PC could be used as a promising anti-cancer therapeutic agent in TNBCs without toxicity to normal cells.

## Additional files

Additional file 1: Figure S1. Phycocyanin induces apoptosis in MDA MB 231 cells. Increased cytochrome C expression after 6 h of PC treatment as analyzed by western blot. (PNG 83 kb)
Additional file 2: Figure S2. Phycocyanin induces apoptosis in MDA MB 231 cells. Phycocyanin (5 μM) treatment at mentioned time points showed cleaved caspase levels. (PNG 60 kb)
Additional file 3: Figure S3. Phcocyanin targets MAPK signaling pathway. Phycocyanin (3 μM) treatment at mentioned time points did not alter the phosphorylation levels of both (A) AKT and (B) NFkB-p65. (PNG 212 kb)
Additional file 4: Figure S4. Phycocyanin is non-toxic to blood cells. Phycocyanin (20 μM) treated (A) RBC’s did not show aberrant morphological changes and (B) Platelet failed to aggregate after 2 h. (PNG 513 kb)

## Abbreviations

PC: Phycocyanin
TNBC: Triple-negative breast cancer
ER: Estrogen receptor
PR: Progesterone receptor
HER-2: Human epidermal growth factor receptor
CDK-2: Cyclin-dependent kinase 2
BAX: bcl-2-like protein 4
Bcl2: B-cell lymphoma 2
MAPK: Mitogen-activated protein kinase
ERK1/2: Extracellular signal related protein kinases 1 and 2
Mcl1: Induced myeloid leukemia cell differentiation protein
COX-2: Cyclooxygenase 2
PGE2: Prostaglandin E2
VEGFR-2: Vascular endothelial growth factor receptor 2
MMP-9: Matrix metallopeptidase 9
