# Supplementary figures and images for: Molecular mechanism of anti-cancer activity of phycocyanin in triple-negative breast cancer cells
Source: BMC Cancer. 2015 Oct 23;15:768. doi: 10.1186/s12885-015-1784-x (PMC4619068; doi:10.1186/s12885-015-1784-x)

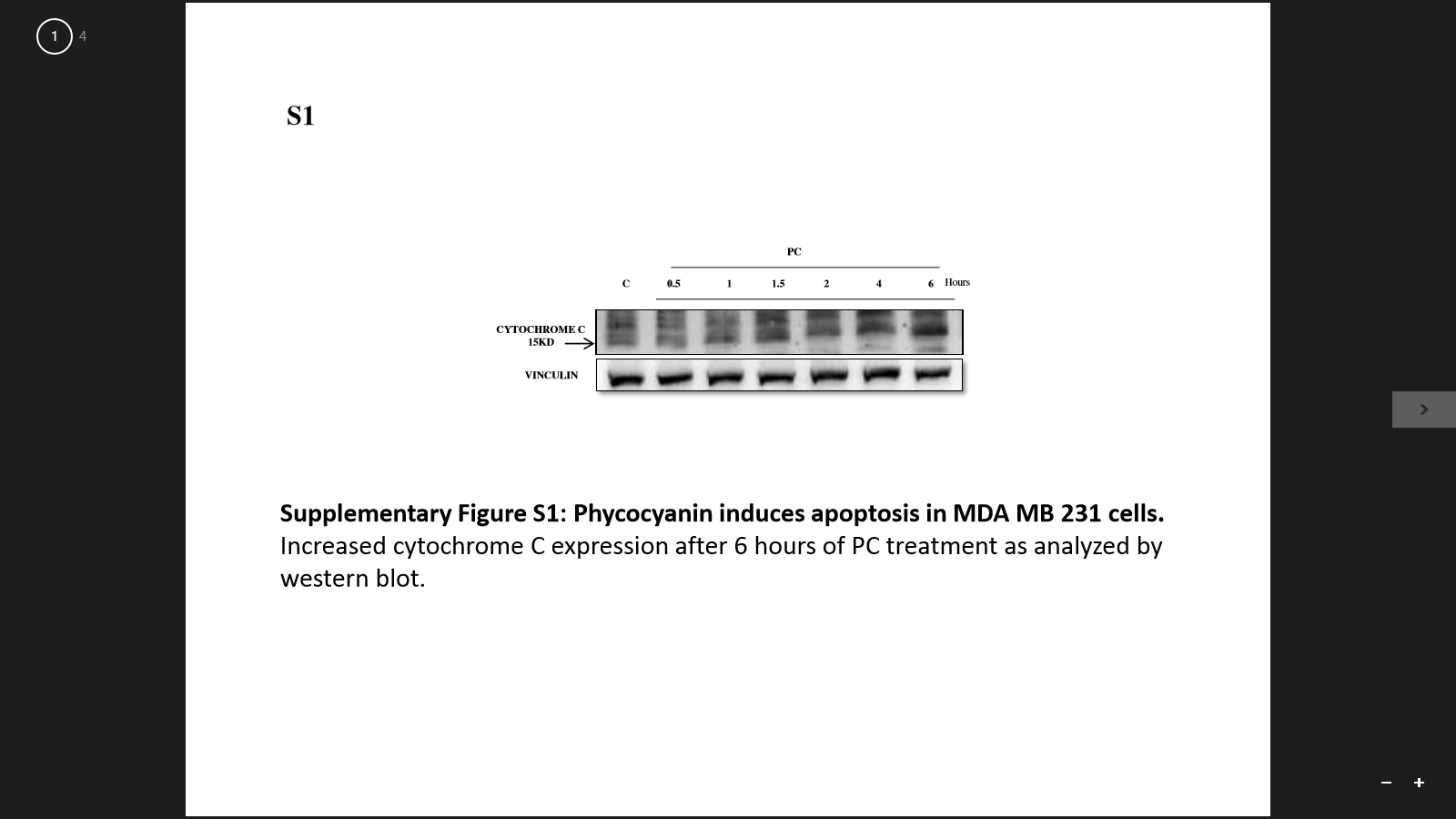

Supplement: Additional file 1: Figure S1. — Phycocyanin induces apoptosis in MDA MB 231 cells. Increased cytochrome C expression after 6 h of PC treatment as analyzed by western blot. (PNG 83 kb) [file 12885_2015_1784_MOESM1_ESM.png]

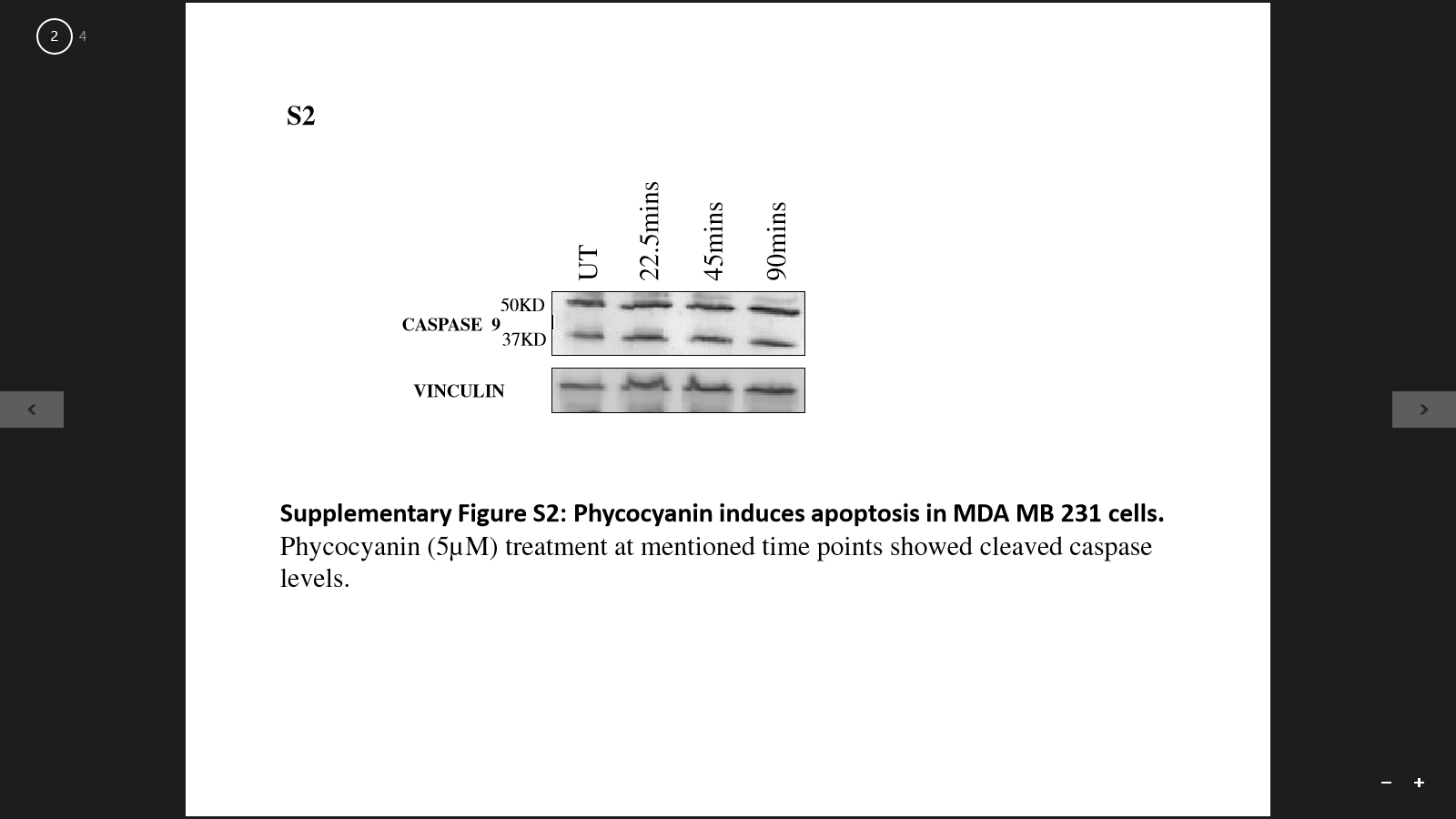

Supplement: Additional file 2: Figure S2. — Phycocyanin induces apoptosis in MDA MB 231 cells. Phycocyanin (5 μM) treatment at mentioned time points showed cleaved caspase levels. (PNG 60 kb) [file 12885_2015_1784_MOESM2_ESM.png]

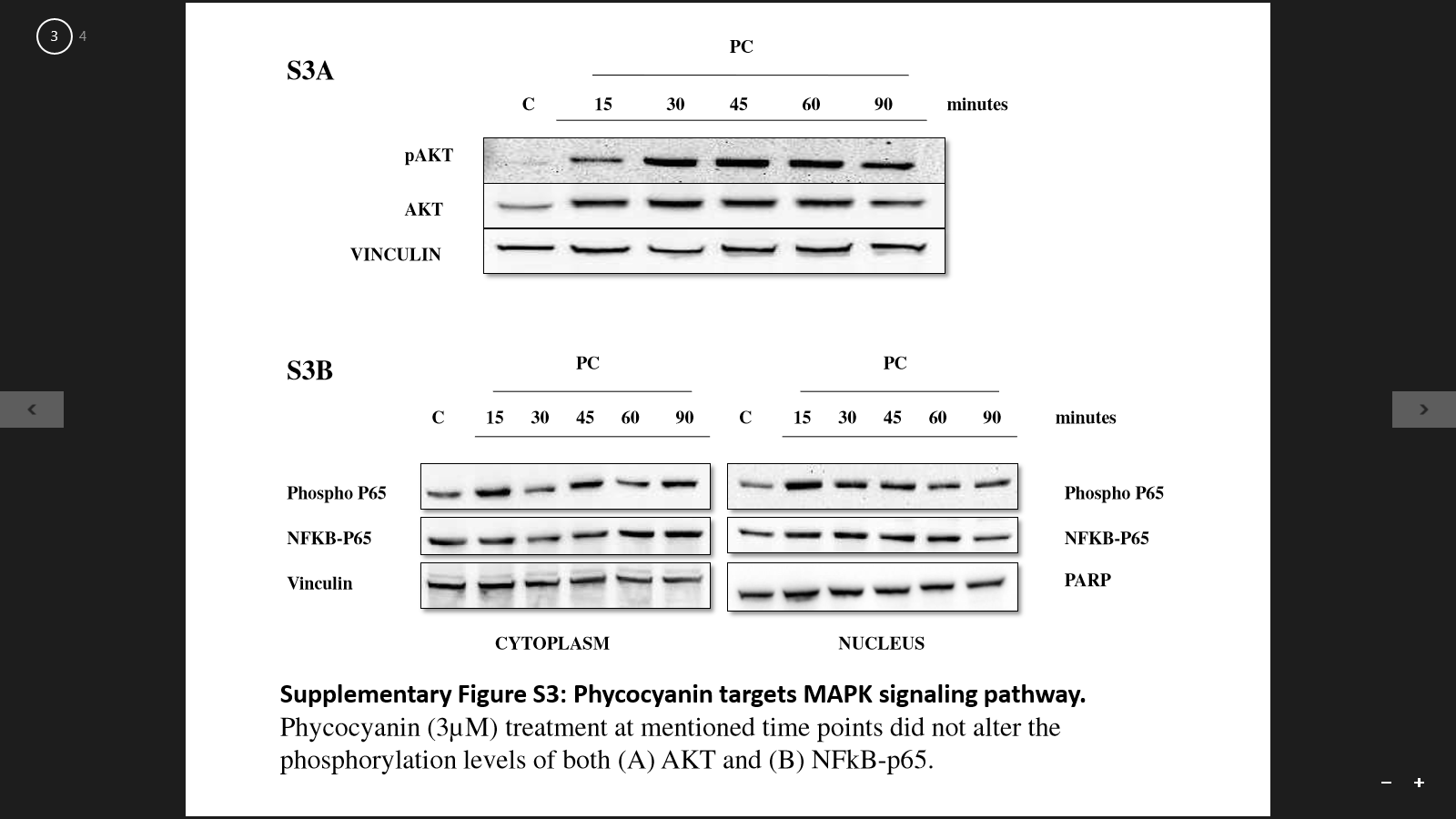

Supplement: Additional file 3: Figure S3. — Phcocyanin targets MAPK signaling pathway. Phycocyanin (3 μM) treatment at mentioned time points did not alter the phosphorylation levels of both (A) AKT and (B) NFkB-p65. (PNG 212 kb) [file 12885_2015_1784_MOESM3_ESM.png]

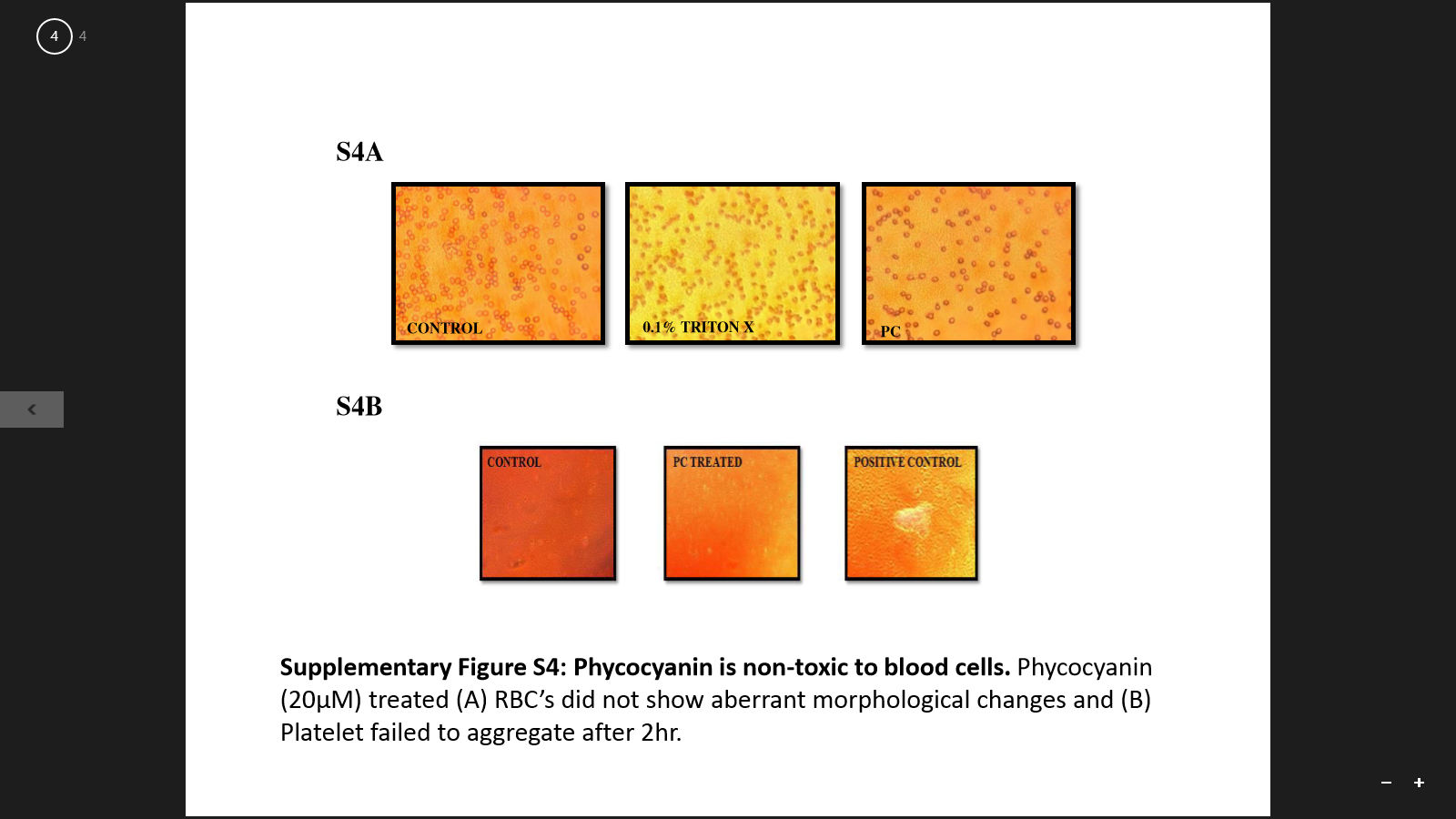

Supplement: Additional file 4: Figure S4. — Phycocyanin is non-toxic to blood cells. Phycocyanin (20 μM) treated (A) RBC’s did not show aberrant morphological changes and (B) Platelet failed to aggregate after 2 h. (PNG 513 kb) [file 12885_2015_1784_MOESM4_ESM.png]
